# The relationship between DNA damage and repair and the occurrence and development of disease

**DOI:** 10.3389/fgene.2026.1849071

**Published:** 2026-06-10

**Authors:** Chongbing Sun, Haimei Li, Fang Xia, Xiaohong Zhang, Xiaoqing Han

**Affiliations:** Weifang People’s Hospital, Shandong Second Medical University, Weifang, China

**Keywords:** cancer therapy, DNA damage, mutagenesis, neoplasms, synthetic lethality

## Abstract

DNA damage can lead to the development of diseases such as tumors. To counteract this, organisms have evolved various repair mechanisms targeting different types of damage. In eukaryotes, there are four main types of DNA repair: nucleotide excision repair (NER), base excision repair (BER), mismatch repair (MMR), and double-strand break repair (DSB repair). NER can remove large fragments of DNA damage, BER can repair damage to individual bases, MMR is used to repair base mismatch, while DSB repair includes two mechanisms: non-homologous end joining (NHEJ) and homologous recombination (HR). NHEJ directly connects the broken ends without a template, while HR uses intact sister chromatids as repair templates. Studying DNA damage and repair mechanisms can pave the way for developing new clinical drugs.

## 1 Introduction

Gorbunova and Seluanov (2005). Studying DNA damage repair helps understand gene mutation mechanisms, the causes of aging and cancer, and can be used to detect environmental carcinogens (Afzal et al., 2019). In 1949, A. Kerner accidentally discovered that microorganisms like *Streptomyces* griseus could reduce mortality when exposed to visible light immediately after UV radiation (Nguyen et al., 2021). Later, numerous microbial experiments confirmed this phenomenon as an inherent DNA damage repair function in many microorganisms, known as photoreactivation. In 1958, R.L. Hill proved that *Escherichia coli* can repair DNA damage caused by ultraviolet radiation even without visible light irradiation (Jiang et al., 2019). He later showed that other microorganisms also have this ability. At that time, this repair function was called “dark resurrection” or “dark repair”. It was later found that dark repair is common in prokaryotes, lower eukaryotes, and higher eukaryotes, including amphibians and mammals. Dark repair was confirmed to include two types: excision repair and replication repair (Takahashi et al., 2015). This discovery provided important molecular biology evidence for the pathogenesis of malignant tumors and introduced DNA damage repair research into the medical field.

## 2 DNA damage type

DNA stores the genetic information necessary for organisms’ survival and reproduction (Kuchlyan et al., 2020). Therefore, maintaining the integrity of DNA molecules is crucial for cells. External and internal factors often damage or alter DNA molecules. Unlike RNA and proteins, which can be synthesized in large quantities, prokaryotic cells generally have only one copy of DNA, while eukaryotic diploid cells have only one pair of identical DNA molecules (Menzel et al., 2020). If DNA damage or changes in genetic information are not corrected, it may affect the function or survival of somatic cells and impact offspring through germ cells. DNA is the only biological macromolecule that can be repaired in cells, reflecting its importance to life (Ma et al., 2017). Conversely, mutation is a common phenomenon in biological evolution, which both opposes and unifies with genetics (Xu et al., 2016). Not all changes in DNA molecules can be repaired, leading to mutation and evolution in organisms (Yang et al., 2022).

**FIGURE 1:**
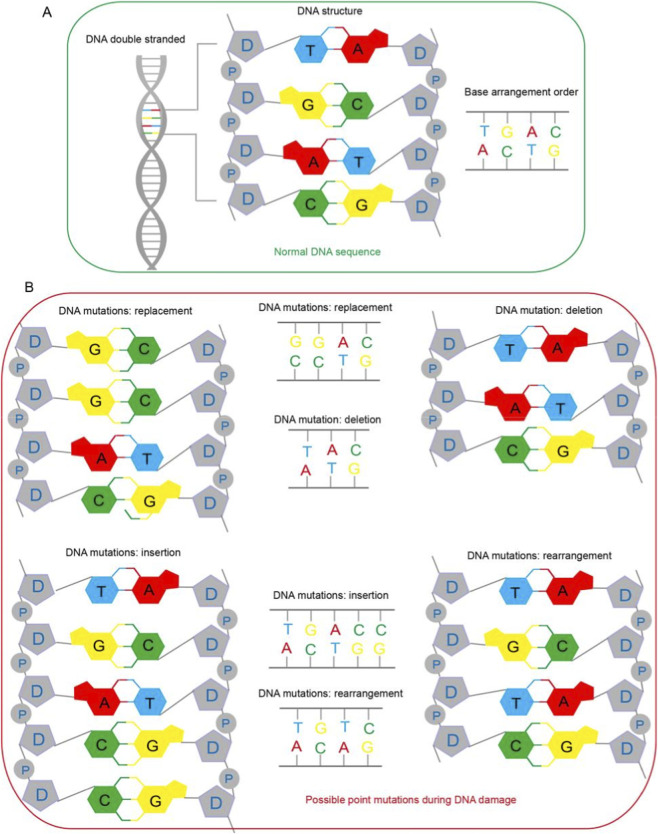
DNA structure and types of mutations after damage. **(A)** DNA is a double helix double strand, composed of a phosphate-deoxyribose backbone and base pairs (A–T, G–C), and the base sequence carries genetic information. **(B)** Types of mutations: Substitution (a single base is replaced by another), Deletion/Insertion (a base is lost or added, potentially causing frameshift), and Rearrangement (a segment of DNA is reversed or relocated).

DNA damage is a phenomenon where the DNA nucleotide sequence undergoes permanent changes during replication, altering genetic characteristics (Zhang et al., 2021). DNA damage can be categorized into substitution, deletion, insertion, and exon skipping (Chakarov et al., 2014). Point mutation refers to the variation of a single base in the DNA sequence (Figure 1). Purine substitution for purine (A and G) and pyrimidine substitution for pyrimidine (C and T) are called transitions. The conversion of purine to pyrimidine or *vice versa* is called transversion (Mukherjee and Vasquez, 2011). Deletion refers to the loss of one or more nucleotides on a DNA strand. Insertion involves adding one or more nucleotides to a DNA strand (Fang et al., 2016). If the number of missing or inserted nucleotides in the coding sequence is not a multiple of three, a reading frame shift occurs, leading to errors in subsequent amino acid sequences. This is called a frameshift mutation (Tronik-Le Roux et al., 2017). Inversion refers to the rearrangement of DNA that reverses a segment or relocates it to a different position. A double-strand break in haploid cells is a lethal event (Tang et al., 2015).

## 3 Causes of DNA damage

### 3.1 Physical damage

**FIGURE 2:**
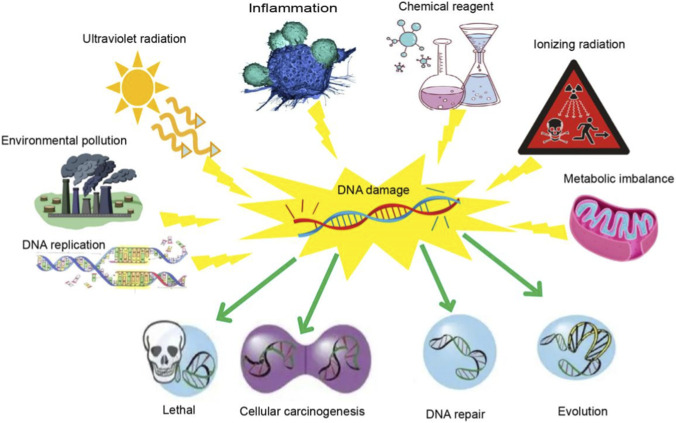
Causes, consequences and associated biological processes of DNA damage. The core “DNA damage” is caused by various factors (ultraviolet radiation, ionizing radiation, chemical agents, inflammation, metabolic imbalance, environmental pollution, and DNA replication errors). The consequences include cell death, carcinogenesis, DNA repair (if successful), and potentially evolution.

Ionizing radiation harms DNA via: 1) the direct effect, where radiation energy directly dissociates the sugar-phosphate backbone; and 2) the indirect effect, where radiation ionizes water molecules to produce reactive oxygen species (ROS), which then chemically attack the DNA bases. Radiation-induced DNA damage is particularly significant. The study of ultraviolet radiation effects revealed the damage it causes to DNA molecules. When DNA is exposed to ultraviolet radiation at its peak absorption wavelength (∼260 nm), it primarily forms covalent bonds between adjacent pyrimidines on the same DNA strand, creating a dimer (Fraikin et al., 2024). Two adjacent thymines (T’s), cytosines (C’s), or a thymine and cytosine can form a dimer through a cyclobutane ring, with thymine dimers being the most common (Nakagawa et al., 2011). In human skin, the frequency of dimer formation due to ultraviolet radiation can reach 5 × 10^4 per cell per hour, but this is limited to the skin since ultraviolet rays cannot penetrate it (D’orazio et al., 2013). However, when microorganisms are exposed to ultraviolet radiation, their survival is significantly affected. Ultraviolet radiation can also cause DNA strand breakage (Fornace et al., 1976). Ionizing radiation induces DNA damage through both direct and indirect effects. The direct effect occurs when DNA absorbs radiation energy directly, leading to its damage, while the indirect effect involves other molecules surrounding DNA (mainly water molecules) absorbing radiation energy, thereby generating highly reactive free radicals that harm DNA (Alizad et al., 2015). Ionizing radiation can induce various alterations in DNA molecules (Ravanat et al., 2001). Firstly, base changes primarily result from OH- free radicals, involving oxidation modification of bases on DNA strands, formation of peroxides, destruction, and shedding of base rings (Huang and Zhou, 2021). Typically, pyrimidine is more sensitive than purine. Regarding changes in deoxyribose, each carbon atom and hydrogen attached to the hydroxyl group of deoxyribose can react with OH-, resulting in the degradation of deoxyribose and ultimately causing DNA strand breakage. DNA strand breakage is a severe consequence of ionizing radiation, and the frequency of broken strands rises with the dosage of irradiation (Stirling et al., 2019). Both the direct and indirect effects of radiation can result in damage to deoxyribose or cleavage of phosphate diester bonds, resulting in DNA strand breakage (Belmar-López et al., 2022). A break in one strand of a DNA double helix is called a single strand break, while a break in both strands at the same or similar location is called a double strand break (Fukuyo et al., 2015). Although single strand breaks occur 10–20 times more frequently than double strand breaks, they are relatively easier to repair. A double strand break in haploid cells (such as bacteria) is lethal. Cross-linking includes DNA strand cross-linking and DNA-protein cross-linking (Tretyakova et al., 2015). Bases on the same or different DNA strands can be covalently linked, and DNA can also be covalently linked to proteins (Ide et al., 2018). Histones, non-histones in chromatin, regulatory proteins, and enzymes related to replication and transcription can all be covalently linked to DNA (Figure 2).

### 3.2 Chemical damage

The initial understanding of chemical factors on DNA damage came from studying the lethality of chemical weapons. Later research on cancer chemotherapy and chemical carcinogenesis increased awareness of the effects of mutagens and carcinogens on DNA. 1. Alkanes damage DNA. Alkanes are electrophilic compounds that easily react with nucleophilic sites of large molecules in living organisms (Jung et al., 2021). Alkylating agents cause various types of DNA damage: (1) base alkylation. Alkylating agents easily add alkyl groups to the N or O of purines or pyrimidines in the DNA strand, with N7 of guanine and N3 of adenine being most vulnerable (Zhu et al., 2021). Alkylation of purine bases changes pairing; for example, alkylated N7 of guanine no longer pairs with cytosine but pairs with thymidine, transforming G-C to A-T. Base shedding occurs (Huang and Zhou, 2021). The glycosidic bond of alkylated guanine is unstable and easily breaks, forming base-free sites on DNA(23). During replication, any nucleotide can be inserted, causing sequence changes. (1) DNA strand breakage The oxygen on the phosphodiester bond of the DNA strand is easily alkylated, forming unstable bonds prone to hydrolysis between sugars and phosphate, causing DNA strand breakage (Kesselring et al., 2020). (2) Crosslinking. There are two types of alkylating agents. One type is a monofunctional alkylating agent, such as methylmethane iodide, which can alkylate only one site; The other type is bifunctional alkylating agents (Zhu et al., 2021). This category includes chemical weapons like nitrogen mustard and sulfur mustard, anti-cancer drugs such as cyclophosphamide, benzoate nitrogen mustard, and mitomycin, and carcinogens like diethylnitrosamine. Their two functional groups can alkylate two sites simultaneously, resulting in cross-linking within DNA strands, between DNA strands, and between DNA and proteins (Poczta et al., 2022). 2. Base analogues and modifiers can damage DNA(33). Some base analogues, such as 5-bromouracil (5-BU), 5-fluorouracil (5-FU), and 2-aminoadenine (2-AP), can be artificially synthesized and used as mutagens or anticancer drugs. Due to its structure being similar to normal bases, it can replace them in the DNA strand and interfere with DNA replication (Çağlayan and Wilson, 2015). For example, the structure of 5-BU is very similar to thymine. It pairs with A in a keto form but can more easily become an enol form pairing with G, leading to A-T converting to G-C during DNA replication (Slater et al., 2015). Certain artificially synthesized or environmental chemicals can specifically modify bases on DNA strands or alter base sequences by affecting DNA replication (Salerno et al., 2021). For example, nitrite can deaminate C to U, and after replication, G-C on DNA can transform into A-T pairs. Hydroxylamine can convert T into C, resulting in the transformation of A-T into C-G pairs (Andrés et al., 2023). Aflatoxin B can also specifically attack bases on DNA, causing sequence changes (Engin and Engin, 2019). These chemicals are carcinogens that induce mutations (Figure 2).

## 4 Consequences of DNA damage

DNA damage includes spontaneous deamination, hydrolysis, various types of breaks, gaps, abasic sites, adducts, inter-chain and intra-chain cross-links, DNA-protein cross-linking, and other subtle chemical modifications (Khanna and Jackson, 2001). DNA damage can hinder accurate replication, controlled transcription, and safe storage of genetic information (Ciccia and Elledge, 2010). Due to numerous exogenous and endogenous genetic toxins, DNA damage continues to occur on a large scale. Some DNA damage is lethal; for instance, damage occurring in germ cells can potentially lead to embryonic death (Zheng and Greenberg, 2018). Lethal mutations within cells may lead to rapid apoptosis. It is estimated that up to 10^5 DNA damages occur in active mammalian cells every day (Hu et al., 2014). Although most of these damages can be effectively removed, some escape detection, cannot be repaired, are repaired too late, or are repaired incorrectly. Over time, DNA damage inevitably accumulates, destabilizing the genome and potentially leading to diseases such as tumors (van den Boogaard et al., 2022). There is a very small probability that DNA damage can promote biological evolution, such as by exposing plant seeds to space radiation to promote evolution (Figure 2).

## 5 The main ways of DNA damage repair

The main methods of DNA damage repair include the following:

Basic excision repair (BER): mainly used to repair single-stranded DNA damage, such as deoxyribonucleotides losing bases or chemically modified bases (Krokan and Bjørås, 2013).

Nucleoside excision repair (NER): mainly used to repair local distortions in DNA double strands, such as pyrimidine dimers caused by ultraviolet radiation. In this process, an enzyme cleaves a small segment of single-stranded DNA from the damaged area. Then, DNA polymerase fills the blank area with the correct base after excision, and finally ligates the newly synthesized fragment to the original DNA (Marteijn et al., 2014).

Mismatch repair (MMR): primarily utilized to correct base pairing errors that occur during DNA replication (Kunkel and Erie, 2015).

Homologous recombination (HR): repair is primarily employed to mend DNA double-strand breaks. During this process, repair enzymes utilize identical sequences found on homologous chromosomes as templates, replacing the damaged section with the correct base sequence through HR (Li and Heyer, 2008).

These repair methods collaborate to preserve DNA stability and integrity.

## 6 The ATM/ATR-p53 signaling axis

DNA damage response (DDR) is coordinated by apical kinases. ATM primarily responds to DSBs, while ATR is activated by single-stranded DNA coated with RPA (Kciuk et al., 2022). Both kinases phosphorylate p53, stabilizing it to induce cell cycle arrest (via p21) or apoptosis (via BAX), thereby preventing the propagation of damaged templates (Kciuk et al., 2022).

## 7 Regulatory signaling pathways related to DNA damage and repair

Double-strand breaks (DSBs) in DNA pose a significant threat to genomic integrity due to their highly toxic nature (Deng et al., 2020). DSBs can be repaired through two main pathways: HR and non-homologous end joining (NHEJ) (Angelina et al., 2022; Puchta, 2004). Numerous studies indicate a close relationship between the selection of repair mechanisms for DSBs and the cell cycle (Branzei and Foiani, 2008). Specifically, during the interphase of mitosis, DSBs are repaired via two mechanisms: HR and NHEJ (Dana et al., 2005; Masahiro et al., 2014); However, these pathways are completely inhibited during mitosis (Dana et al., 2005). Cells can carry DSBs from interphase into mitosis, and DSBs can also form during mitosis due to increased replication pressure (Yang et al., 2019). Cells deficient in HR are prone to accumulating mitotic DSBs, especially under enhanced replication pressure. Consequently, the repair of DSBs during mitosis is an urgent biological issue requiring immediate attention. Research indicates that during the mitotic phase, the broken ends of DSBs can be tethered together by a complex, allowing them to persist through mitosis until the next interphase for repair (Thompson et al., 2019; Blackford and Stucki, 2020). Some researchers suggest that a repair pathway for DSBs, distinct from HR and NHEJ, may be activated during mitosis (Kakarougkas and Jeggo, 2014). Studies have found that the terminal junction function of Pol θ may play a significant role in repairing DSBs, which is crucial for the survival of HR-deficient cells. Therefore, researchers believe that Pol θ may be involved in the DSB repair pathway during mitosis (Beagan et al., 2017). Knockdown of core HR factors (BARD1, BRCA1, PALB2, and BRCA2) significantly decreases the number of Polθ-located DSB loci (Patterson-Fortin and D'Andrea, 2023; Wang et al., 2019). Additionally, IP-MS found an interaction between Polθand these factors. Pol θ mediates Theta-mediated end joining (TMEJ) by identifying microhomologies (usually 2-6 bp) at the flanking sequences of DSBs (Ramsden et al., 2022). It possesses unique helicase and polymerase activities that remove 3′-flaps and bridge the DNA ends, serving as a critical backup pathway when HR is deficient (Azam et al., 2025).

DNA single-strand break repair is a type of DNA damage repair that fixes damage on a single DNA strand. This process is crucial for maintaining genome integrity and genetic stability. During DNA replication, if a damaged DNA region is encountered, the newly synthesized strand will be shorter than the undamaged strand because the damaged site cannot serve as a template. To repair this damage, the complete mother strand will recombine and exchange with the daughter strand with gaps, allowing the corresponding fragments on the mother strand to fill these gaps. DNA polymerase then catalyzes the synthesis of a new fragment to fill the gap on the mother strand. Finally, DNA ligase connects this new fragment with the old strand to complete the repair process (Wienholz et al., 2017; Callegari and Kelly, 2016). This repair mechanism was initially discovered in *E. coli* (Galli et al., 2017). For recA strains that lack repair ability due to their inability to recombine genetically during conjugation, this repair mechanism is called recombinant repair (Fallon, 2021).

In addition, there are various DNA repair mechanisms, including excision repair, light repair, nucleotide excision repair, and base excision repair, each responsible for different types of DNA damage (Zhu et al., 2018). Excision repair, for example, is an important and effective mechanism involving specific endonucleases, pol I, and DNA ligases to repair single-stranded DNA damage, and the loss of deoxyribonucleotides or chemical modification of bases (Krokan and Bjørås, 2013; Zharkov, 2008). Light repair primarily addresses pyrimidine dimers caused by ultraviolet radiation (Grossman et al., 1988).

Overall, DNA single-strand break repair is a crucial process for the accurate transmission of genetic information. It involves the coordinated action of multiple enzymes to ensure timely and precise DNA damage repair.

In humans, the BER pathway is significantly more specialized than in prokaryotes. It is bifurcated into short-patch BER (SP-BER) and long-patch BER (LP-BER) (Xue et al., 2025). While prokaryotic BER primarily involves DNA Polymerase I, human cells utilize DNA polymerase β (Pol β) to replace a single nucleotide in SP-BER (Li et al., 2022). For complex lesions, LP-BER is recruited, involving FEN1 (flap endonuclease) and PCNA (proliferating cell nuclear antigen) to replace a sequence of 2–13 nucleotides (Gohil et al., 2023). The entire process in eukaryotes is coordinated by the scaffold protein XRCC1, which has no direct ortholog in bacteria (Moncan et al., 2023). DNA glycosylase cleaves the bond between the nucleoside base and ribose, leaving the ribose phosphate chain intact but producing apurinic or apyrimidinic (AP) sites (Gembka et al., 2007). 8-Oxoguanine DNA glycosylase I (Ogg1) removes 7,8-dihydro-8-oxoguanine (8-oxoG), a mutation caused by reactive oxygen species (D’augustin et al., 2020). Uracil DNA glycosylase, another BER enzyme, cleaves uracil, the deamination product of cytosine, to prevent subsequent C-T point mutations (Manapkyzy, 2024). N-Methylpurine DNA glycosylase (MPG) removes various modified purine bases (Adhikari et al., 2009).

**FIGURE 3:**
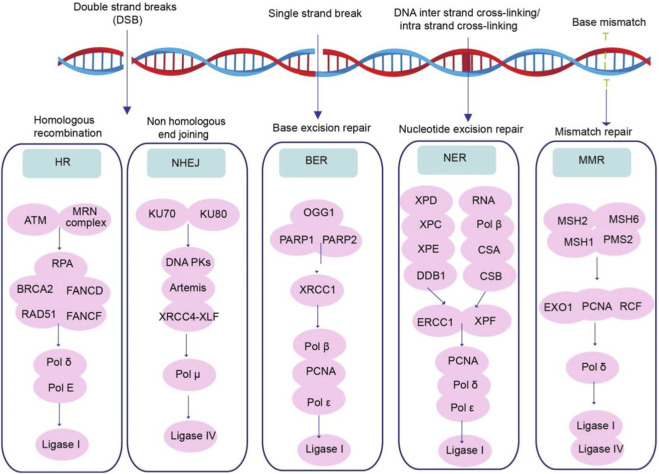
Major DNA repair pathways and their key molecules. Different types of DNA damage (double-strand breaks, single-strand breaks, cross-links, base mismatches) are repaired by distinct pathways: HR (BRCA1/2, RAD51) and NHEJ (KU70/80, DNA-PKcs, XRCC4, LIG4) for DSBs; BER (OGG1, APE1, Polβ, XRCC1, LIG3) for base lesions; NER (XPC, XPA, XPG, ERCC1-XPF) for bulky adducts; and MMR (MSH2/6, MLH1/PMS2) for replication errors.

AP sites generated by the BER enzyme action in DNA, and by depyrimidine and depurine reactions, can be repaired by AP endonuclease 1 (APE1) (Maher et al., 2019). APE1 cleaves the phosphodiester chain 5′to the AP site (Maher et al., 2019). The DNA strand contains a 3′-hydroxyl group and a 5′-alkyl deoxyribonucleic acid. DNA polymerase β (Pol β) inserts the correct nucleotide based on the corresponding Watson-Crick pairing and removes deoxyribonucleic acid via its AP lyase activity (Prasad et al., 2016). X-ray repair cross-complementing protein 1 (XRCC1) is necessary for forming heterodimers with DNA ligase III (LIG3) (Liu et al., 2023). As a scaffold protein, XRCC1 provides a non-active binding site for Pol β and binds Pol β and LIG3 together at the repair site (Pe et al., 2006; Parsons et al., 2005). Poly(ADP-ribose) polymerase (PARP-1) interacts with XRCC1 and Pol β, forming an essential component of the BER pathway (Lebedeva et al., 2026). The final repair step is completed by LIG3, which connects the replaced nucleotide’s deoxyribose to the deoxyribose phosphate backbone. This approach is called “Short Patch Error Rate” (Bhagwat et al., 1999). Another approach, known as “long patch repair,” involves replacing a nucleotide chain with a minimum length of 2 nucleotides (Roy, 2023). Reports indicate that the repair length ranges from 10 to 12 nucleotides (Sancar, 1996). BER requires the presence of proliferating cell nuclear antigen (PCNA), which acts as a scaffold protein for recombinases (Kalweit, 2025; Strzalka and Ziemienowicz, 2011). DNA polymerase enzymes, possibly Pol δ and Pol ε, generate oligonucleotide flaps (Hindges and Hübscher, 1997). Flap endonuclease-1 (FEN1) removes the existing nucleotide sequence (Ouanounou, 2023). DNA ligase I (LIG1) then connects the oligonucleotides to the DNA, blocking the cleavage and completing the repair process (Turco et al., 2013) (Figure 3).

Human NER is distinct from the prokaryotic UvrABC system and consists of two sub-pathways: Global Genome NER (GG-NER), which scans the entire genome for lesions using the XPC-RAD23B complex, and Transcription-Coupled NER (TC-NER), which is activated when RNA polymerase II is stalled (van Toorn et al., 2022). In humans, the damaged DNA segment (typically 24–32 nucleotides) is excised through the coordinated action of endonucleases XPG (3′incision) and the ERCC1-XPF complex (5′incision) (Gohil and Roy, 2024). This contrasts with prokaryotes where a much shorter fragment is removed by the UvrABC excinuclease (Wozniak and Simmons, 2022). It primarily repairs pyrimidine dimers, structurally large carcinogen-DNA adducts, and various damages causing DNA helix distortion. In this repair system, there are two pathways: the whole genome repair GG-NER pathway, which can repair DNA damage throughout the genome, characterized by slow speed (Kuper and Kisker, 2023); The second pathway is the Transcriptional Coupled Repair (TCR) pathway, which mainly repairs various DNA damages hindering the extension of RNA polymerase, characterized by relatively fast repair speed (Hanawalt, 1994; Hanawalt, 2002). These two approaches share similar repair processes after identifying damage. Environmental carcinogens can enter the human body, causing DNA damage (Barnes et al., 2018). If the body cannot repair the damaged DNA promptly and accurately, it may lead to genetic mutations, resulting in tumor occurrence.

DNA damage can arise not only from cells exposed to genetic toxins but also from abnormal DNA processing. The DNA repair pathway for replication-related errors is termed MMR (Caccese et al., 2020). During DNA synthesis, MMR corrects nucleotide misintegration to prevent permanent DNA alterations in dividing cells (Reyes et al., 2020).

Unlike the methyl-directed MMR in *E. coli* (mediated by MutH and Dam methylase), human cells lack DNA methylation for strand discrimination. Instead, they rely on PCNA loading and pre-existing nicks (such as Okazaki fragments) to identify the nascent strand (On, 2022). Mismatch recognition in humans is performed by two specialized heterodimers: MutSα (MSH2-MSH6) for single base mismatches and MutSβ (MSH2-MSH3) for small insertion/deletion loops (Jayaraj et al., 2023). These complexes then recruit MutLα (MLH1-PMS2) to initiate the excision process, a mechanism crucial for preventing Microsatellite Instability (MSI) in human cancers (Reitsam et al., 2022). During DNA replication, the N6 position of A in the GATC sequence of the template strand undergoes methylation. The newly synthesized strand shows a gradient of methylation, with the least methylation near the replication fork. Thus, the newly synthesized DNA double-stranded molecule is in a semi-methylated state (Koppenhöfer et al., 2019). MMR system utilizes this principle by using the template chain’s base as a guide and correcting the mismatched nucleotide on the daughter strand. The mismatch repair system in prokaryotes like *E. coli* is encoded by the Mut S gene (Hasenauer et al., 2025). Mut H and Mut L proteins, comprising Mut S and Mut L, repeat once per protein to form a tetramer, Mut SL, which slides along the DNA(106). When a bulge occurs due to base mismatch, the process halts and initiates the pulling of the double-stranded DNA from both sides into a loop until the GATC sequence is identified. If N6 of A undergoes methylation, indicating it as a parent strand that remains uncut, whereas if A of GATC is not methylated, indicating it as a daughter strand, Mut H binds to Mut SL (Zhang et al., 2024). Mut H possesses endonuclease activity, cleaving at the 5′end of the GATC sequence, specifically on the left side of G, followed by DNase I’s exonuclease activity to eliminate all loops twisted out of the Mut SL tetramer (Au et al., 1992), DNA POL III and DNA ligase are subsequently refilled. In eukaryotes like humans, the genes coding for Mut are hMSH2 and hMLH1, which correspond to Mut S and Mut L. In humans, the functional mismatch recognition is performed by heterodimers MutSα (MSH2-MSH6) for single-base mismatches and MutSβ (MSH2-MSH3) for small insertions/deletions (Kaur and Badhe, 2025). The recruitment of MutLα (MLH1-PMS2) initiates the downstream excision. Defects in these proteins lead to Microsatellite Instability (MSI), a hallmark of Lynch syndrome and certain sporadic cancers (Krause, 2025). The remaining steps of the process remain unchanged, with detailed gene coding products present in mismatch repair genes (Figure 3).

## 8 DNA repair and human disease

### 8.1 Cancer and genomic instability

MMR defects resulting from genetic mutations or epigenetic silencing may elevate the occurrence of spontaneous mutations, often linked to hereditary and sporadic cancers. Tomasetti C and colleagues found that two-thirds of the mutations found in 17 types of cancer were caused by DNA replication errors (Tomasetti et al., 2017). The polymorphism of the human OGG1 gene is associated with an increased risk of various cancers, including lung and prostate cancer (Wang et al., 2025). DNA damage can induce genetic alterations, and if it affects genes regulating cell growth, such mutations can predispose to cancer. Beyond cancer, DNA damage accumulation also plays a critical role in aging and neurodegenerative diseases, as discussed below.

### 8.2 Aging and neurodegenerative diseases

There is evidence to suggest that damage to nuclear DNA (encoding most cellular RNA and proteins) and mitochondrial DNA is associated with aging (Kowalska et al., 2020). DNA damage can also trigger cell death, with profound implications for the organism harboring the affected cells, such as the irreplaceable loss of neurons in the brain (Wong and Chow, 2023). The accumulation of DNA damage is also deemed as one of the factors contributing to aging (Soto-Palma et al., 2022). In addition to age-related pathologies, inherited defects in DNA repair machinery directly cause a group of human genetic disorders, which are summarized in the following section.

### 8.3 Hereditary DNA repair deficiency syndromes

Cellular DNA damage has been associated with the development of numerous human diseases, including pigmented keratosis, amyotrophic lateral sclerosis (ALS), Bloom syndrome, and Werner syndrome (Lindor et al., 2000). In 1968, American scholar J.E. Clifford discovered that Xeroderma pigmentosum (XP), an autosomal recessive inherited photochemical cancer disease in humans, is caused by DNA damage from genetic mutations and defects in excision repair (Brambullo et al., 2022).

## 9 Discussion

Accurate reading of DNA sequences is essential to express functional messenger RNA in cells and ultimately generate proteins. Faithful DNA replication during cell division is crucial for daughter cells to inherit complete genetic material from the mother cell (Selak et al., 2008). Various changes in cellular DNA caused by internal and external factors can have profound biological consequences. Although most DNA damage can be repaired, the repair system’s efficiency is not 100%. Unlike nuclear DNA, mitochondrial DNA (mtDNA) lacks NER and primarily relies on a specialized form of BER (Bazzani et al., 2022). Due to its proximity to the respiratory chain (a source of ROS) and less efficient repair compared to nuclear DNA, mtDNA is highly susceptible to oxidative damage, contributing significantly to degenerative diseases and aging (Bazzani et al., 2022).

Naturally, mutations are not permanent adversaries. Imagine if DNA repair mechanisms were thorough enough to rectify all DNA damage without causing mutations; in such a scenario, genetic alterations and evolutionary substrates would not exist. Depending on circumstances, DNA damage can be either pathogenic or therapeutic.

Although human genomic DNA is frequently damaged, most of it can be successfully repaired by various mechanisms. If DNA lesions are not repaired promptly or are too severe to repair, they can trigger signaling events leading to three possible cell fates: aging, apoptosis, or cancer (van den Boogaard et al., 2022).

Although DNA damage is crucial in the development and evolution of cancer cells, sustained damage is used in clinical cancer treatment to induce apoptosis or senescence in malignant cells (Roos et al., 2016). Chemotherapy drugs like bleomycin, mitomycin, and cisplatin are effective because they further damage the DNA of cancer cells, which replicate faster than surrounding tissues (van den Boogaard et al., 2022). The cellular DNA repair mechanism is a double-edged sword. While it reduces mutations that may lead to cancer by maintaining genomic integrity, in malignant cells, it allows survival despite additional DNA damage, leading to uncontrolled growth. To block this survival mechanism in cancer cells, clinical trials are testing inhibitors targeting specific DNA repair enzymes, including MGMT, (poly(ADP-ribose) polymerase)PARP, and DNA-PK (Drew et al., 2025).

Many anti-tumor drugs are closely linked to DNA damage and repair. Classic chemotherapy drugs, such as platinum and topoisomerase inhibitors, affect DNA replication and directly cause DNA damage (Nitiss, 2009); New ADC drugs also use cytotoxic agents to directly cause DNA damage and recognize specific antigens on tumor cell surfaces through linked recombinant monoclonal antibodies, allowing for more precise targeting of tumor cells (Jin et al., 2022). DDR-inhibiting drugs, such as PARP1 inhibitors that target key proteins in the DDR pathway, kill tumor cells through synthetic lethality mechanisms. PARP1 inhibitors exploit the principle of synthetic lethality in BRCA-deficient cells, where the loss of both BER (inhibited by PARP inhibitor) and HR (due to BRCA mutation) leads to irreparable DSBs and cell death (Kumar, 2025). However, clinical resistance often arises through secondary ‘reversion mutations’ in BRCA genes that restore HR function or via increased drug efflux (Jia et al., 2024). Additionally, abnormal DDR can alter the tumor immune microenvironment, directly affecting the efficacy of ICIs. These drugs have significantly improved the treatment efficacy and survival rates of lung cancer patients (Wei et al., 2021). Based on this, the above-mentioned drugs have been explored for combined therapy in lung cancer using a synergistic mechanism (Xiong et al., 2022). The efficacy of a new combination therapy model, involving PARP1 inhibitors combined with chemotherapy or ICIs, is of great concern (D'Andrea, 2018; Hunia et al., 2022). However, current studies show that except for PARP1 inhibitors combined with temozolomide improving the objective response rate (ORR) in first-line treatment for Small cell lung cancer (SCLC), there is no significant improvement in patient efficacy in first-line and maintenance therapy for advanced SCLC, nor in combinations of PARP1 inhibitors with chemotherapy or ICIs (Shen et al., 2022; Wanderley et al., 2022). These combinations now offer survival benefits to patients in first-line treatment of advanced NSCLC. Deficiency in mismatch repair (dMMR) results in a high Tumor Mutational Burden (TMB), leading to the production of numerous neoantigens (Sun et al., 2025). This high-TMB state enhances the tumor’s visibility to the immune system, explaining the superior efficacy of Immune Checkpoint Inhibitors (ICIs) in MSI-H/dMMR patients.

## 10 Conclusion

DNA damage repair is a fundamental guardian of genomic stability. While various pathways specialize in distinct lesions, their dysregulation leads to cancer, aging, and neurodegeneration. Future therapies must focus on overcoming resistance to DDR inhibitors and leveraging repair deficiencies to enhance immunotherapy.
